# Agricultural Subscribers

**Published:** 1920-10-30

**Authors:** 


### Agricultural Subscribers.

WE are glad to see that the scheme to organise help in the country parishes that form the agricultural industry round about Ipswich for the benefit of the East Suffolk and Ipswich Hospital is proving successful. Mr. Arthur Griffiths, the Secretary of the Hospital, lately took the chair at a joint conference of the Farmers' Union, the Workers' Union, and the Agricultural Labourers' Union; the representatives were all in favour of a weekly system of contributions from farmers and their men, and this after the members of the organisations had been approached.

Mr. Griffiths and Mr. Clement Smith made a good point when explaining that the hospital served 250 parishes, which would probably represent 75,000 souls, or a population approximately the same as that of the borough of Ipswich. The workers in the town had been very well organised, and generously gave last year £4,350. If the countrymen could be organised on the same scale (they subscribed last year £830), the maintenance of the hospital would probably be secure.

It was therefore resolved to send a joint letter, signed by the officers of the unions, employers, and employed, to all members in support of the scheme; and to appoint in each parish by the Farmers' and Workers' Unions an independent person to collect the money and transmit it periodically to the hospital. For the present the scheme is to apply to that part of the county shown on the map published in the annual report, which depicts the area defined many years ago between the hospitals. Later, when success is assured, the scheme, by arrangement, can be extended to the north and west of Suffolk.

